# Nicotinic alpha 7 receptor expression and modulation of the lung epithelial response to lipopolysaccharide

**DOI:** 10.1371/journal.pone.0175367

**Published:** 2017-04-06

**Authors:** Lorise C. Gahring, Elizabeth J. Myers, Diane M. Dunn, Robert B. Weiss, Scott W. Rogers

**Affiliations:** 1 Geriatric Research, Education, and Clinical Center, Salt Lake City Veterans Administration Medical Center, Salt Lake City, Utah, United States of America; 2 Division of Geriatrics, Department of Internal Medicine, University of Utah School of Medicine, Salt Lake City, Utah, United States of America; 3 Department of Human Genetics, University of Utah School of Medicine, Salt Lake City, Utah, United States of America; 4 Department of Neurobiology and Anatomy, University of Utah School of Medicine, Salt Lake City, Utah, United States of America; Emory University School of Medicine, UNITED STATES

## Abstract

Nicotine modulates multiple inflammatory responses in the lung through the nicotinic acetylcholine receptor subtype alpha7 (α7). Previously we reported that α7 modulates both the hematopoietic and epithelium responses in the lung to the bacterial inflammogen, lipopolysaccharide (LPS). Here we apply immunohistochemistry, flow cytometry and RNA-Seq analysis of isolated distal lung epithelium to further define α7-expression and function in this tissue. Mouse lines were used that co-express a bicistronic tau-green fluorescent protein (tGFP) as a reporter of α7 (α7^G^) expression and that harbor an α7 with a specific point mutation (α7^E260A:G^) that selectively uncouples it from cell calcium-signaling mechanisms. The tGFP reporter reveals strong cell-specific α7-expression by alveolar macrophages (AM), Club cells and ATII cells. Ciliated cells do not express detectible tGFP, but their numbers decrease by one-third in the α7^E260A:G^ lung compared to controls. Transcriptional comparisons (RNA-Seq) between α7^G^ and α7^E260A:G^ enriched lung epithelium 24 hours after challenge with either intra-nasal (i.n.) saline or LPS reveals a robust α7-genotype impact on both the stasis and inflammatory response of this tissue. Overall the α7^E260A:G^ lung epithelium exhibits reduced inflammatory cytokine/chemokine expression to i.n. LPS. Transcripts specific to Club cells (e.g., CC10, secretoglobins and Muc5b) or to ATII cells (e.g., surfactant proteins) were constitutively decreased in in the α7^E260A:G^ lung, but they were strongly induced in response to i.n. LPS. Protein analysis applying immunohistochemistry and ELISA also revealed α7-associated differences suggested by RNA-Seq including altered mucin protein 5b (Muc5b) accumulation in the α7^E260A:G^ bronchia, that in some cases appeared to form airway plugs, and a substantial increase in extracellular matrix deposits around α7^E260A:G^ airway bronchia linings that was not seen in controls. Our results show that α7 is an important modulator of normal gene expression stasis and the response to an inhaled inflammogen in the distal lung epithelium. Further, when normal α7 signaling is disrupted, changes in lung gene expression resemble those associated with long-term lung pathologies seen in humans who use inhaled nicotine products.

## Introduction

The progression of a multitude of cellular responses that govern normal and pathological processes are modulated by nicotine through its interaction with ionotropic nicotinic acetylcholine receptors (nAChR, [1–5]). In terms nAChRs, they contribute to complex tissue responses such as to inflammogens through coordinate and cell specific signaling by diverse cell-types. These cell types range from neuronal cells such as those involved in parasympathetic function to non-neuronal cells including those of hematopoietic cells such as macrophages, keratinocytes of the skin, and lung epithelium [3,6–9]. One of the more prominent nAChRs through which effects are imparted is the nAChR subtype alpha7 (α7). In this context the α7 response to nicotine generally suppresses the overall inflammatory response. This can be demonstrated in the α7^KO^ mouse which exhibits an exaggerated peripheral response to the inflammogen LPS, but it lacks the normal suppression by nicotine [2,3,8,10]. The mechanism of α7 signaling is in part related to its unique channel properties that in addition to causing membrane depolarization (as on neurons and similar to other nAChRs), includes an exceptionally large calcium current that is sufficient to activate multiple down-stream targets including Creb, NfκB, Jak/Stat and PI3K pathways [4,11]. Thus a better understanding of the tissue- and cell-specific mechanisms modulated by α7 could improve the pharmacological targeting of anti-inflammatory agents that is already being tested and increase the potential of this receptor as a more specific target in clinical applications [1–5].

The mouse model of α7-inflammatory interaction is of considerable value towards understanding how this receptor impacts cellular responses. To better understand these mechanisms, we used a genetic approach [12–14]. Through homologous recombination, mice were constructed in which a bi-cistronic IRES-driven tau:green fluorescent protein (tGFP) extension of the native α7 transcript provides a reporter of receptor gene transcription (α7^G^; [12]). In this background a precise point mutation was introduced to change the glutamic acid 260 to an alanine and specifically limit the relatively high calcium current through this receptor (α7^E260A:G^; [4,14–16]). This effectively uncouples the α7 from calcium signaling mechanisms with minimal perturbation to genomic context or other receptor functions. Further, a reliable genetic model is produced in which α7 receptor mediated calcium-directed cell-specific pathways induced in response to inflammatory and immune stimuli can be defined.

Pharmacological agents specific to α7 suppress the response to LPS [7,10], a result that was confirmed and extended in the lung of α7^E260A:G^ mice [14]. In the α7^E260A:G^ mouse the inflammatory response by both hematopoietic and local non-hematopoietic cells to intranasal (i.n.) LPS was overall significantly decreased [14]. Also notable was the reduction of inflammatory cell infiltration into the α7^E260A:G^ lung as measured in the bronchial alveolar lavage fluid (BALF) despite the relatively normal recruitment of inflammatory cells into the blood from the bone-marrow. Notably, in reciprocal chimeric mice constructed from bone marrow of differing α7 genotypes it was shown that the non-hematopoietic cells of the α7^E260A:G^ mouse lung dominated this overall inefficient LPS-response and the poor recruitment of bone-marrow derived inflammatory cells to the lung [14]. This study extends those findings to examine the α7^E260A:G^ CD45^-^ resident cell transcriptional responses to i.n. LPS. This includes defining the cells that express α7 transcripts in the lung and their transcriptional responses to i.n. LPS through applying RNA-Seq based comparisons. Immunohistochemical localization of the tGFP as a reporter of α7-expression in both α7^G^ and α7^E260A:G^ adult distal lung tissue revealed strong expression predominantly by CD45^+^ alveolar macrophages and in CD45^-^ Club (Clara) and ATII epithelium cells. The α7^E260A:G^ mice exhibit an increase in Club cells (identified by Scgb1a1 (CC10)) and a corresponding decrease in ciliated cells (as visualized by acylated-α-tubulin). The CD45^-^ epithelial cells were enriched and examined by RNA-Seq interrogation which revealed altered lung epithelial stasis and inflammatory response in the α7^E260A:G^ mouse. This includes differences in inflammatory cytokines such as IL-1β and TNFα and some chemokines (e.g. Cxcl10), which failed to elevate in the α7^E260A:G^ mouse after i.n. LPS. Other notable changes in the lung CD45^-^ cell transcript response to this inflammogen included dramatic shifts in Scgb1a1 (CC10), Muc5b, surfactant proteins (Sftpa1, Sftpb and Sftpc, respectively), and other genes of regulatory importance including Bpfia1 (Splunc1) and Bpifb1 (Plunc1). This transcriptional difference was accompanied by the accumulation of Muc5b protein in distal bronchia and bronchiole airway spaces that is present only in the α7^E260A:G^ mouse. Another particularly notable shift in transcript profiles in the α7^E260A:G^ CD45^-^ lung cells included increased expression of genes involved in fibrotic processes such as collagen and associated processing metalloproteases and binding proteins. Masson’s trichrome staining of the α7^E260A:G^ lung, independent of LPS challenge, confirmed the occurrence of substantially increased fibrotic deposits in these mice. These results extend our earlier findings of a relationship between signaling through α7 and normal lung cell function in response to exogenous inflammogens. Thus, the dysfunction of the α7 receptor, as may occur during exposure to ligands including nicotine, could have severe consequences to long-term pulmonary health.

## Materials and methods

Animals. In all cases animals were maintained according to the Guide for the Care and use of Laboratory Animals of the National Institutes of Health and in accordance with protocols approved in advance by the Institutional Animal Care and Use Committee at the University of Utah (Protocol Number #12–06001) and the VA Medical Center (Protocol A14/17), Salt Lake City, UT. Mice were housed in pathogen free cages with standard mouse chow and water provided ad libitum with 12 hour light/dark cycling. Groups of 5 mice were age, gender and strain matched for each experiment. The details regarding the construction of the α7^G^ and α7^E260A:G^ mouse lines using the precision of homologous recombination was described previously [12–14]. The strategy used insures that the normal gene copy number, genomic context and expression regulatory elements of α7 transcription and protein are retained. The reporter of α7 transcript expression is a tau:GPF fusion protein (tGFP), which is expressed as an independently translated protein from a bi-cistronic extension of the α7-transcript harboring an intervening IRES element [12]. The α7^E260A:G^ point mutation was generated in the α7^G^ background and has been described in detail [14]. This also permits α7^G^ to serve as a genetically-matched control to α7^E260A:G^.

Intranasal (i.n.) administration of LPS. Intranasal LPS (i.n. LPS) administration is a minimally invasive procedure used to stimulate lung inflammatory responses and has been described [14]. Briefly, after complete sedation, using intraperitoneal (i.p.) injection of an anesthetizing dose of tribromoethanol (Avertin, Sigma, St. Louis, MO) in sterile saline, mice (5 per group) received either i.n. *E*. *coli* lipopolysaccharide (LPS 055:B5; Sigma, St. Louis, MO) at 250 μg/mouse in 30 μl of saline (15μl/nostril) or for control mice an inoculum of 30μl saline (15 μl/nostril). To confirm a positive response to LPS, at 18 hrs post-LPS treatment (i.n.) mice were quickly bled (50 μl of heparinized blood) and this blood analyzed for Gr1^+^CD11b^+^ by Flow Cytometry. Mice exhibiting a minimum 30% increase in circulating Gr1^+^CD11b^+^ cells in both α7^G^ and α7^E260A:G^ mice were used. At 24 hrs after this LPS challenge mice were sacrificed by injection of a lethal dose of tribromoethanol (Avertin) and bronchial alveolar lavage fluid (BALF) was collected by gently flushing lungs (5–6 times) with 1 ml of buffer (DPBS, 2% BSA, 0.05% EDTA) via insertion of a butterfly needle into the trachea. These cells were used to assess responsiveness to LPS by flow cytometry. Following the lavage to remove BALF cells, the lung tissue was diced and incubated with dissociation medium (10 ml DMEM, 500 μl DNase 1 (1 mg/ml) containing 100 μl Liberase (2.5 mg/ml; Roche) in a water bath at 37°C for 40 min. Halfway through this incubation, this interstitial tissue prep was gently passed through an 18-gauge needle. Cells were centrifuged at 900xg, at 4°C for 10 min., followed by RBC lysis and washes with buffer. Trachea and esophagus were excluded from harvested tissue prior to dissociation.

Enrichment of CD45^+^ and interstitial CD45^-^ cells. Isolated interstitial cells [14] were pelleted by centrifugation, the supernatant aspirated and the cells resuspended in 90 μL of running buffer (0.2% FBS, 2mM EDTA in 1X PBS) per 10^7^ cells. Microbeads coupled to an anti-CD45 antibody (Miltenyi Biotec, San Diego, CA) were added at a concentration of 10 μL microbeads per 10^7^ cells as per manufacturers recommended protocol. Cells were mixed by brief vortexing and incubated for 15 minutes at 4°C. Cells were washed by adding 5 ml running buffer and centrifuged. The supernatants were aspirated and the cell pellets were resuspended in 2 mL of buffer. Cell suspensions were passed through a 70 μm nylon mesh filters prior to separation. Interstitial CD45^+^ and CD45^-^ cells were isolated using an autoMACS cell separator (Miltenyi Biotec). Isolated CD45^-^ fractions were routinely tested for purity (routinely 92–95%) and determined for viability which was routinely high (less than 3–5% 7-AAD (viability stain) positive [13,14].

Transcriptome profiling by RNA-Seq analysis and PCR validation. RNA from the CD45^-^ cell population was prepared from the microbead enriched cell fraction as described previously [13,14]. The concentration and purity of the RNA was determined with a ND-1000 Spectrophotometer (NanoDrop Technologies, Wilmington, DE). Strand-specific, RNA-Seq libraries were generated using Illumina TruSeq stranded mRNA preparation kits. Libraries were then sequenced on an Illumina HiSeq 2500 sequencer at a depth of 40 million reads per sample. Tophat and STAR [17,18] were used to align RNA-Seq reads to the mouse genome (UCSC Genome; Assembly NCBI37/mm9). CDS read counts were extracted from bam files using the *htseq-count* script [19] features defined by mouse annotation files such as the GENCODE gene sets [20]. Also, the RSEM package [21] was another approach used to quantify transcript abundances from RNA-Seq data. Differential transcript expression was evaluated using the edgeR package within the Bioconductor project [22]. Gene list analysis suggestive of physical and co-expression interactions was performed using the GeneMANIA analysis program and database [23,24]. Confirmation of RNA-Seq results using quantitative TaqMan polymerase chain reaction (qPCR) were conducted as described previously [13,14].

Immunohistochemistry. Mouse lungs were prepared for immunohistochemical analysis as described [25]. Briefly, mice were lethally anesthetized, exsanguinated by severing the abdominal aorta and the chest was opened. The trachea was cannulated and lungs filled with 4% paraformaldehyde/2.5% sucrose for 10 minutes. After this time the cannula was removed and the lungs were carefully dissected out. Lungs were post-fixed in 30% sucrose, embedded in tissue embedding media in tissue blocks, and 10 μM frozen sections cut with a cryostat. Sections of the large lobe were analyzed. Antibodies used for these experiments were to the immunogens: aquporin5 (Aqp5, 1:250, goat, Santa Cruz biotechnology); acylated-α-tubulin (acTub, 1:250, mouse, Santa Cruz biotechnology), CC10 (Clara cell 10, 1:500, rabbit, Millipore), Calcitonin gene-related peptide (CGRP; 1:300, rabbit, Immunostar); green fluorescent protein (GFP, 1:800; Chicken, Aves); ionized calcium-binding adapter molecule 1 (Iba1, 1:750, rabbit, Wako); Muc5b (1:500, mouse, Abcam), Peripherin (1:500, rabbit, Abcam); Protein gene product 9.5 (PGP 9.5, 1:500, rabbit, Abcam); tyrosine hydroxylase (1:500, Rabbit, Chemicon); neuropeptide Y (NPY, 1:250, Rabbit, Novus). All secondary antibodies (donkey) of appropriate specificity and fluorochromes or chromophore conjugation were obtained from Jackson ImmunoResearch.

## Results

The lung is composed of cell types whose functions include structural and physiological support to ongoing respiration but also initiating the appropriate immune responses to inhaled foreign materials such as bacteria [26,27]. We have extended our earlier findings to identify those cells in the lung that express α7 by applying immunohistochemistry to detect the co-expressed tGFP reporter protein (Methods).

### Immunohistochemical analysis reveals expression of α7 in the lung is restricted to distinct cell subpopulations

The robust expression of tGFP is readily detected and it corresponds well with α7-expression [12,28]. Further, the genetic-reporter approach avoids concerns pertaining to the sensitivity and specificity of immune-reagents directed towards direct detection of the α7 receptor (e.g.,[29]). In the mouse, and similar to humans, the identity and distribution of cells lining the airways differ in a proximal-distal direction depending upon the lung sub-compartment (e.g., trachea, bronchi and bronchioles and alveoli [30–34]). This analysis focused on cells of the distal lung lining including bronchial passages and alveoli and excluding the trachea. In these regions the expression of the tGFP reporter of α7-expression was detected in subpopulations of CD45^+^ (bone marrow derived) cells including macrophages, neural and neuro-epithelial cells as well as CD45^-^ non-neuronal interstitial cells including the epithelium as described in detail below.

### Macrophages

**Fig 1:**
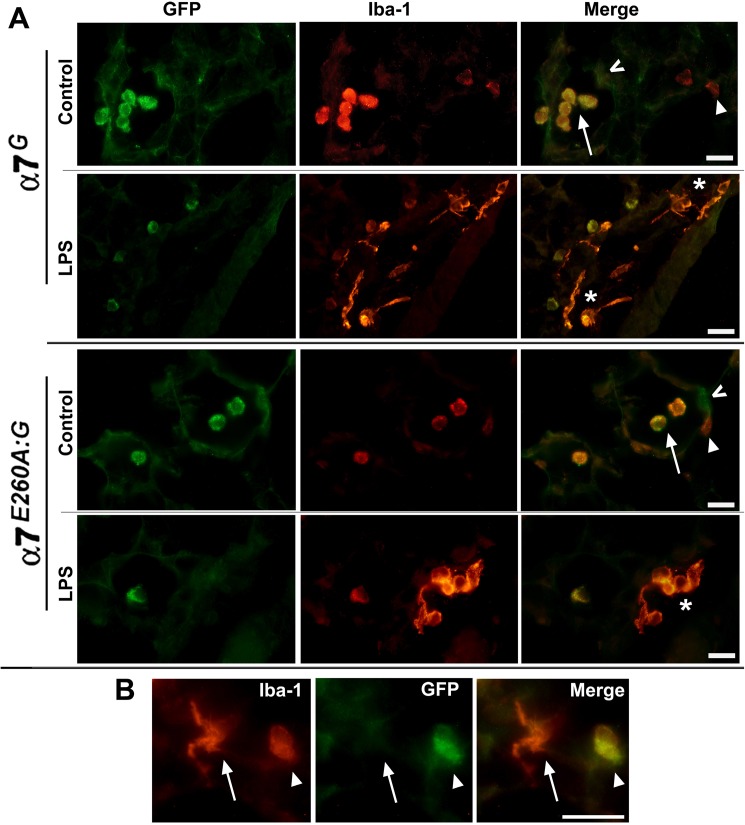
Immunostaining for α7^G^ tGFP reporter expression in lung immune cells co-expressing Iba-1. A) In both the α7^G^ and α7^E260A:G^ mouse tGFP is produced as a bicistronic reporter of α7 transcript expression. Immunohistochemistry of the lungs from these animals reveals strong GFP expression by alveolar macrophages (AM; arrow) that are co-labeled by Iba-1 (red) in the control (saline) or i.n. LPS treated lungs of both α7-genotypes. Also present in interstitial regions adjacent to alveoli are Iba-1 labeled cells that are not co-stained for tGFP expression (arrow head). These cells exhibit a more flat in morphology and reduced Iba-1 signaling compared to AMs. In lungs 24 hours after i.n. LPS exposure, cells strongly labeled by Iba-1 of amoeboid-like morphology are evident (asterisks). These prominent cells suggestive of activated macrophages do not co-label with tGFP. AMs are also present in both α7-genotypes as identified by co-expression of Iba-1 and tGFP, their location in alveolar spaces and the retention of their spheroid morphology. Other cells in the alveolar lining also labeled with tGFP (**^** and see Fig 3). B) Increased magnification of an α7^G^ control alveolar region identifies two Iba-1 staining macrophages of differing morphology that are either within or associated in part with the interstitial region (arrow) or only in the alveolar space (arrow head). Co-expression of tGFP reveals α7-expression only by the AM cell. Results were similar in the α7^E260A:G^ lung (not shown). Bars = 30 microns.

Macrophages of the lungs reside in both the parenchymal tissue and the bronchial and alveolar spaces. To determine if these cells express α7, we co-labeled lung tissue sections to identify the α7-expression reporter (anti-GFP antibody) with an antibody to Iba1. The Iba1 marker was selected because it is present on both AM and interstitial macrophages. Other markers such as CD11c, which is present on AM, and CD11b [13,14] which is expressed by interstitial macrophages have also been used with results consistent with Iba1 staining and location of cells (not shown). Iba1 intensely co-stained with tGFP in mice of both α7 genotypes (Fig 1) which is notable in that staining is effectively restricted to alveolar macrophages (AM). In parallel sections the identity of these cells as AMs was further supported by the absence of co-labeling with CD11b (not shown). Interstitial (parenchymal) macrophages (IM; Iba1+) in normal lung sections were confirmed by CD11b co-staining, but these cells do not exhibit co-staining with tGFP over background (not shown, but see Fig 1A and 1B).

The lung airway inflammatory response to i.n. LPS is characterized in both the α7^G^ and α7^E260A:G^ mice by an influx of macrophages into both the alveolar space and interstitial tissue [35]. These cells are strongly labeled by Iba1, they exhibit a much flatter amoeboid morphology (Fig 1A) and they are positive for CD11b^+^ expression (not shown). Together this is consistent with their identity as either resident interstitial macrophage (IM) or bone marrow infiltrating macrophages and they lack detectable tGFP immunostaining (Fig 1A) indicating they do not express transcripts for α7. An increase in the infiltration by other cell types such as granulocytes, T and B cells or other immune cells relative to unstimulated lung tissue at this relatively early time following i.n. LPS challenge is not observed consistent with our previous report ([14]; not shown). Alveolar macrophages retain tGFP immunoreactivity in LPS treated mice and their morphology does not appear different from AM in non-stimulated lung (Fig 1A). Thus, two distinct classes of macrophages are distinguished in the lung based upon their co-expression of detectable tGFP and Iba1 (Fig 1A and 1B), where AMs strongly co-express tGFP but IM macrophages, or those of a more amoeboid-morphology expected of activated macrophages following i.n. LPS exposure, do not express this α7-reporter.

### Neuronal and neuroepithelial

**Fig 2:**
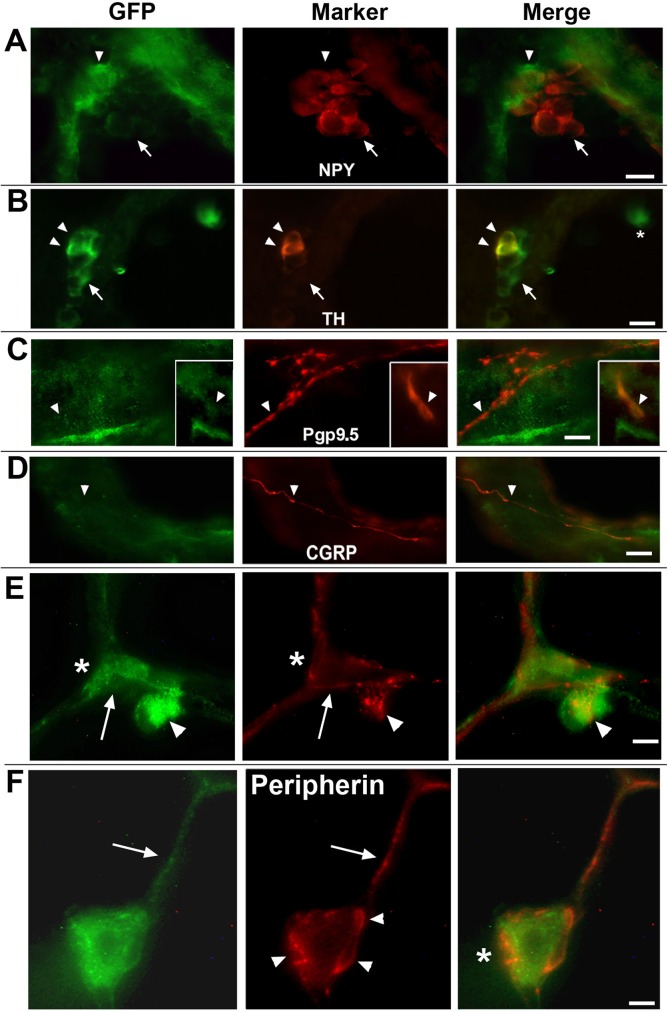
Immunohistochemical examination of the α7 reporter by lung neuronal and neuroepithelial cells. Panels A through F show images of the α7 reporter tGFP co-expression in lung sections co-stained with various neuronal and neuroepithelial markers. A) Expression of neuropeptide Y (NPY) is observed occasionally in infrequent cell clusters associated with brachial epithelium as reported by others [37]. When present, cells reactive for GFP were adjacent to (arrow head), but did not overlap with, cells expressing NPY (arrow). B) Tyrosine hydroxylase (TH) identifies cells capable of catecholamine synthesis usually in association with neuroendocrine functions. These cell clusters were in part identified by TH immunostaining (arrow heads) and localization to sites of bronchial bifurcation consistent with their identity as Pulmonary neuroepithelial bodies [38] and they contained a mix of GFP stained cells (arrow) and cells co-expressing TH (arrow heads). C) Protein-G product 9.5 (Pgp9.5, aka; ubiquitin C-terminal hydrolase, Uchl1) immunofluorescence in lung sections labeled nerve fibers (arrow head) and occasional solitary cells (inset, arrow). No co-expression of this marker with tGFP was observed. D) Calcitonin-gene related peptide (CGRP) labeled lung sensory fibers did not exhibit detectable tGFP co-expression. E,F) Sensory innervation of the lung by peripherin labeled fibers was prevalent and as shown were particularly prominent in processes of the alveolar compartments that did not label with GFP (arrow). Some of these processes may interact with tGFP-labeled ATII cells (asterisk; see Fig 3). The interaction between AMs that are in contact with alveolar cell linings and peripherin labeled processes is notable (arrowhead). This includes the appearance of specialized neuronal process end points that form a complex of interactions with these stationary AMs. F) Increased magnification shows a strongly tGFP labeled AM (arrows) that is in association with the alveoli and are richly associated with peripherin positive fibers (arrowheads). These process endings often wrap the AMs and multiple process swellings that are interacting with the AM (arrowheads) and in some cases form indentations on the AM (right panel, asterisk). Bars = A-E, 15 microns; F, 5 microns.

The lung is a rich source of neuroepithelial and neuronal cells (and their autonomic processes) [36]. This includes cells that are identified by detection with antibodies directed towards neuropeptide Y (NPY), tyrosine hydroxylase (TH), pgp9.5, CGRP and peripherin. Cell subtypes of the distal lung that express the tGFP reporter of α7 transcripts include neuroepithelial (NE) cells (Fig 2A and 2B). NPY expression in respiratory epithelium was limited to infrequent cell clusters associated with bronchial linings (Fig 2A). These cells did not co-express tGFP. However, in all cases at the base of the NPY-identified cells were several additional cells that were strongly labeled for anti-GFP immunostaining but lacked detectable NPY signal. The identity of these tGFP^+^/NPY^-^ cells remains to be determined although a role in response to inflammatory challenge by similar cells located at the base of NPY clusters has been discussed [37]. More common were cells labeled by TH (Fig 2B). These elongated solitary cells were found along the bronchial lining and in cell groupings that formed small clusters near points of bronchial bifurcation. These clusters are consistent with neuroendocrine bodies (NEB; [36,38]). The co-expression of tGFP with TH localized to a limited subset of the NEB cells at the base of clusters. Those TH labeled cells extending towards the more distal aspect of the cluster did not co-express TH indicating heterogeneity in these cell phenotypes. The TH labeled solitary cells did not co-express tGFP (not shown). TH-labeled cells were observed only in bronchial passages and not in the alveoli as anticipated from previous reports regarding the distribution of these cells in the lung [39,40].

In addition to cell bodies labeled by tGFP, there is considerable labeling of neuronal processes including those associated with the extended vagal nerve in the basal structures surrounding the bronchi and those proceeding into the alveoli (Fig 2C–2E). The detection of axon nerve fibers is made possible by the transport of tGFP into these processes as part of the tau-protein fusion partner to GFP [12]. The markers examined included those that identify neuroendocrine and proprioceptive neurons including Ppg9.5 (Fig 2C) or CGRP (Fig 2D), respectively, and did not co-label with tGFP. Innervation of NEBs (at their basal aspect) by vagal nerve afferents [36,41,42] also were labeled by these markers but they did not co-express tGFP (not shown, but see Fig 2B). This also appears to be the case for the majority of processes labeled by antibodies to peripherin, a Type III intermediate filament protein associated with slow-conducting nociceptive C-fibers (Fig 2E). One striking aspect of the peripherin-labeled axons is their frequent association of what appear to be specialized axon termini and AMs that are associated with airway cell linings (Fig 2E and 2F). These interactions are particularly common in the α7^E260A:G^ mouse, but relatively infrequent in the α7^G^ control. At increased magnification these termini appear to outline and even indent the interacting AM cells suggestive of a direct association (Fig 2F). Notably, at this magnification the co-expression of tGFP by these peripherin-labeled processes is suggested, although this remains to be confirmed. The interaction between these fibers and AMs is reminiscent of other reports that show direct interactions between innervating purinergic fibers and alveolar macrophages in the lung [43] and similar to that observed in other tissues as well [44]. No clear demonstration of similar interaction between tGFP-labeled neuronal processes and IMs was observed, however this possibility cannot be ruled-out and it will require additional analysis to assure the extent of any additional functionally relevant α7 neuronal-immune cell interactions.

### Epithelium

**Fig 3:**
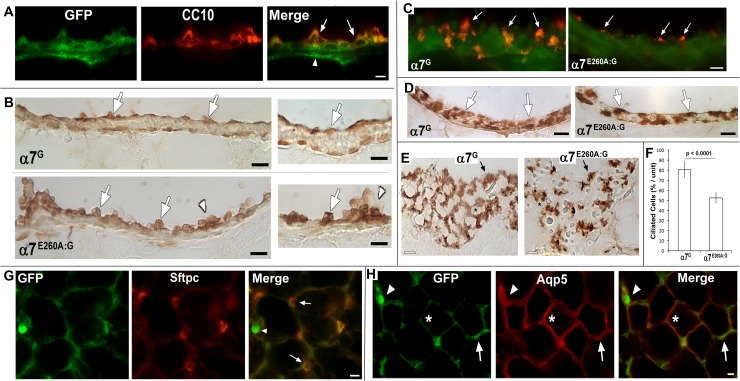
tGFP (GFP) expression by major lung cell subtypes. A) The definitive Club cell marker, CC10, is shown to co-localize with GFP (arrows). Other GFP staining in the bronchial lining includes neuronal axons (arrow head; see Fig 2). Bar = 15 microns. B) Examination of Club cell anti-CC10 staining revealed by a peroxidase stained section of both α7^G^ and α7^E260A:G^ distal bronchial sections is shown at two magnifications. The panels to the right show greater magnification. In the lower magnification images (panels to the left) the typical appearance of the relatively smooth surface of the α7^G^ bronchia and including evenly distributed Club cells (arrows) is observed. In contrast, the α7^E260A:G^ bronchial lining is more uneven and Club cells appear to protruded from the bronchial surface (arrows). In the α7^E260A:G^ lung this includes aggregate-like clusters (arrowheads) of these cells that are rare or absent from the α7^G^ lung. This is particularly evident in the images of increased magnification to the right. The CC10-labeled Club cells (arrows) are evident and again in the α7^E260A:G^ commonly protrude into the airway space. Relatively large aggregates (arrow heads) are also evident. Bars = 25 microns (left panels) and (right panels) = 15 microns. C) Merged images of immune-localization of GFP (green) and acylated-α-tubulin (acTub, red) shows ciliated cells (arrows) typical of α7^G^ (left panel) or in the α7^E260A^ (right panel) lung. No co-expression of tGFP and acTub was observed. Also the ciliated cells of the α7^E260A^ mouse appeared to be fewer and of altered morphology relative to the control. Bar = 15 microns. D) Sections similar to those from (C) but stained using peroxide to reveal acTub in ciliated cells. Consistent with fluorescent immunostaining, ciliated cells are relatively evenly dispersed in the α7^G^ lung bronchial linings and prominent well-defined cilium are present. In the α7^E260A:G^ lung similar staining shows ciliated cells that tend to be dispersed further apart that often lack well defined cilia (arrows). E) A longitudinal section reveals the bronchial surface facing the airway and the distribution of acTub stained ciliated cells (arrows). Their distribution and the cilium complexity differ between these α7-genotypes and the regular networks formed by ciliated cells (asterisk) in the α7^G^ lung are absent in the α7^E260A:G^. Bar = 15 microns. F) Quantitation of ciliated cell number comparing the α7^G^ to the α7^E260A:G^ mouse. The average percent of ciliated cells per unit measure was made from 5 sections from each of 3 mice of the identified α7 genotype. A highly significant difference shows that α7^E260A:G^ lung harbors approximately one-third fewer ciliated cells relative to the α7^G^ lung. G) Co-expression of GFP and the ATII cell marker, surfactant protein c (Sftpc; arrows). The arrow head identifies an AM. H) The GFP staining is compared to aquaporin 5 (Aqp5), a marker of ATI cells (asterisk). Essentially no overlap in expression between these markers was observed. An ATII cell is identified by an arrow and an AM by an arrowhead. Staining for these cells was essentially identical between α7^G^ (shown) and α7^E260A:G^ mice with differences to be discussed. For G and H the Bars = 15 microns.

The CD45^-^ cells of the lung include epithelium, neuro-epithelium, endothelium, and fibroblasts [45]. These cells also respond to inflammatory stimuli and they contribute to the overall immune response. Mouse distal lungs harbor a particular enrichment of Club, ciliated, alveolar type I and type II cells and reduced overall goblet and basal cells in this region [26,46]. As shown in Fig 3, tGFP immunostaining is detected strongly in Club cells as identified by the co-expression of the cell-specific marker, CC10 (Fig 3A), consistent with other reports [25]. When peroxidase-based staining is used to identify CC10 expressing cells, a clear comparison between distal epithelium of the α7^G^ with α7^E260A:G^ lung is revealed. This includes a difference in their morphology and distribution (Fig 3B). The α7^E260A:G^ Club cells appear to be more bulbar and they protrude into the airway space more than observed in the α7^G^. Further, these cells are often seen to form irregular clusters that are not observed in the control α7^G^ (Fig 3B).

The expression of tGFP by other cell types associated with the bronchial epithelium lining was not detected. This included immunostained sections to reveal the expression of acTub, a marker of ciliated cells (Fig 3C), and the more infrequent basal and goblet cells (located near proximal bronchial sites; not shown). Although ciliated cells do not express detectable tGFP, their frequency and distribution in the bronchial epithelium lining differed markedly between the α7^G^ and α7^E260A:G^ (Fig 3C and 3D). Ciliated cells were less frequent and appeared to be less ciliated than similar cells in sections from the α7^G^ cells. This difference in distribution was particularly evident in sections of the lung prepared at an angle that reveals their occurrence along bronchial surfaces (Fig 3E). Again, in the α7^E260A:G^ the ciliated cells appear to be reduced in number and the cilia associated with them less evident than the prominent cilia of the α7^G^. Also, there is an absence of the networking of these cells along the surface facing the airway that in the α7^G^ resembles a more honeycomb-like lattice. The appearance of fewer ciliated cells in the α7^E260A:G^ was confirmed by stereological-based counts of multiple lung sections from 5 mice (5 sections per mouse) of each genotype (Fig 3F). The results show the α7^E260A:G^ bronchia harbors approximately one-third fewer ciliated cells when compared with the α7^G^, a result that is highly significant.

In contrast to the conducting airways, cell type diversity in the lining of alveoli are composed of essentially only squamous alveolar type I (ATI) cells and the more cuboidal ATII cells [26,46]. In the adult, ATI cells cover approximately 90% of the alveolar surface and interact closely with endothelial cells of pulmonary capillaries to facilitate gas exchange. In contrast the less frequent and more cuboidal ATII cells express surfactant proteins and are found interspersed among ATI cells [26]. This functional diversity offers the ability to distinguish them based upon the expression of several markers such as aquaporin 5 (Aqp5) to identify ATI cells, or several surfactant proteins for ATII cells. In this study alveolar cells labeled by surfactant protein C (Fig 3G) also co-labeled with tGFP confirming strong expression of this reporter of α7-expression by ATII cells. No co-expression of tGFP was detected in ATI cells identified by Aqp5 staining (Fig 3H). Thus, ongoing α7-tGFP expression is predominantly limited to two specific cell subtypes in the distal lung epithelium which includes Club and ATII cells (see also [14]).

### The epithelial transcriptional response to LPS is modified by α7

**Fig 4:**
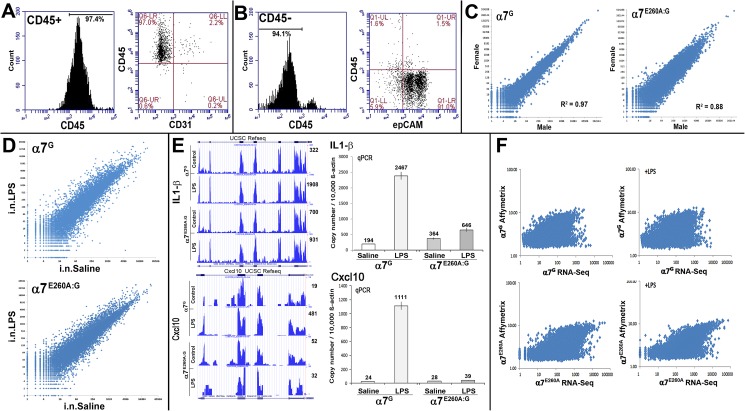
Isolation of CD45^-^ lung epithelium and characterization of RNA transcript expression. A) BALF was collected from groups of 3–5 mice, pooled, and blocked with anti-CD16/CD32 antibodies (FcRγ) to prevent non-specific binding. These cells were then stained with monoclonal antibodies directed against mouse CD45 (marker of bone marrow derived cells) and CD31 (endothelial cell marker) and analyzed using flow cytometry. In the BALF 97% or more of the cells were CD45+. B) The lung tissue was removed, trachea and proximal structures removed by dissection, and the remaining tissue (interstitium) was digested. These single cell suspensions Fc-blocked and stained as above and described in the Methods. Stained samples were analyzed using flow cytometry. More than 94% of the cells were CD45^-^ and greater than 90% were epCam positive defining epithelial cells. C) Cells from CD45^-^ fractions were then collected for each α7-genotype, RNA prepared and RNA-Seq performed (Methods and text). The results show a log(2) plot comparison of transcript expression between the α7g male-female or α7^E260A:G^ male female analyses. Linear regression (R^2^) of the results demonstrates high correspondence between the respective genders of each genotype. D) Comparisons of RNA-Seq results (Log(2) plots) between CD45^-^ distal lung cell fractions from α7^G^ or α7^E260A:G^ genotypes both following exposure to intranasal saline (control) or i.n. lipopolysaccharide (i.n.LPS). E) Examples of RNA-Seq results for two genes (interleukin1-β (IL-1β) and chemokine Cxcl10) overlaid with the UCSC RefSeq. The read coverage graphs taken from the UCSC browser reflect the results of raw counts (total) per sample. Because the library sizes for each sample were similar, differences in magnitude do not account for shifts in the reported relative transcript values (not shown). The quantitation (shown adjacent to the plot for each sample) after different treatments is also compared with the results of qPCR analysis for these gene transcripts. Note the overall agreement between these measures that was typical for these results. Further, neither the IL-1β or Cxcl10 gene transcript in the α7^E260A:G^ lung CD45^-^ fraction was responsive relative to the substantial increase in transcription in the control following LPS exposure. F) Plots comparing the result of RNA samples analyzed by RNA-Seq versus the Affymetrix based array platform. Overall good agreement between these methods was achieved although the expected compression of the dynamic range in Affymetrix samples is evident.

Previously we found that α7^E260A:G^ mice respond to i.n. LPS (as measured by elevated numbers of inflammatory cells in the blood) but fail to recruit hematopoietic cells into the lung, a result that was associated with altered transcriptional signaling profiles by non-hematopoietic resident lung cells [13,14]. To increase the resolution of how α7-expression impacts the CD45^-^ cell-specific response to LPS, we compared the α7 and α7^E260A:G^ distal lung epithelial transcriptional response to i.n. saline or i.n. LPS using a polyadenylated transcriptome strategy combined with RNA-Seq quantification. This approach is greatly facilitated by two key experimental factors. First, the α7^E260A:G^ mouse was constructed in the α7^G^ background to provide a genetically defined experimental system in which the α7-coupled response by specific cell subtypes to inflammatory challenge can be directly measured. Second, the detection of a cell-specific impact by α7 is greatly enhanced by the ability to enrich cells of a desired phenotype using specific markers (Methods). This is particularly important in the lung where the contribution by CD45^+^ cells of bone marrow origin can dominate the detection of often more subtle changes in the transcriptional response to i.n. LPS by other cell types such as those in the epithelium. As before [14], mice were treated with 250 μg LPS/mouse in 30 μl saline and control mice received only 30 μl i.n. saline. The systemic response to LPS was confirmed by removing a small blood sample to measure the influx of inflammatory cells into the blood (Methods). Upon confirmation of an LPS response, the mice were sacrificed 24 hours following i.n. LPS, the lungs cleared of BALF content by lavage, the distal lung tissue enriched by dissection and removal of the trachea and the remaining interstitial cells dissociated to facilitate the selective enrichment of epithelial cells. To do this the CD45^-^ cells were collected from the flow-through of a CD45^+^ magnetic bead column that routinely produced >90% enrichment after a single pass (Fig 4A). Of the enriched CD45^-^ cells the majority (>95%) were epCAM+ (Fig 4B), a marker defining epithelial cells including Club cells, ciliated cells, ATI and ATII cells. Further analysis revealed the samples used contained few endothelial cells (<1% CD31^+^; not shown, but see Fig 4A). The epithelial enriched population was then extracted, poly-adenylated RNA collected and these samples were processed further for analysis by RNA-Seq (Methods). Tests of reproducibility and the possible contributions from gender effects were examined by comparing different experimental samples and samples taken from male and female lung epithelium of both α7 mouse genotypes (Fig 4C). This analysis revealed highly significant correspondence in the results between both independently replicated preparations as well as between genders (Y-chromosome specific transcripts were removed). For example, the expression correlation between α7^G^ male and female was highly significant (R^2^ = 0.97). The α7^E260A:G^ mouse gender comparisons were also highly significant albeit slightly reduced correlation (R^2^ = 0.88). The results demonstrated that in addition to attaining reproducibility between independent samples, significant differences among transcripts in α7 and α7^E260A:G^ mice associated with gender were not evident.

Examples of the RNA-Seq profiles of CD45^-^ lung transcriptomes from control and LPS treated α7^G^ or α7^E260A:G^ mice are shown in Fig 4D. To better diminish interference by system noise and false-positive signals produced from minor transcripts whose small differences tend to become amplified, the RNA-Seq data were converted to quantitation of coding sequence (CDS) data (Methods). This method offered several advantages. First, there is excellent agreement between the transcript read-data and the UCSC reference sequence. This further assures a greatly reduced and frequent high false-positive rate and a high confidence in reproducibility between samples. This was particularly important for comparisons of the results from LPS responses where differences among pro-inflammatory cytokines and chemokines can be obscured in the absence of CDS refinement. Examples of this include IL-1β or Cxcl10. In the α7^G^ cells the response of IL-1β is a 6-fold increase and for Cxcl10 a 25-fold increase but in the α7^E260A:G^ mouse this is reduced to 1.3-fold and 0.61-fold, respectively (Fig 4E). Also largely removed using this approach were false-positive reads that mapped to the 3’-UTR and other repetitive elements located in intergenic and intron regions. To further validate these measurements, we used real-time PCR using Taqman gene probes (quantitative PCR; qPCR) to measure the gene transcription levels independently for a subset of genes that exhibit varied expression (Fig 4F). Even in epithelial cells for lesser-expressed transcripts such as Cxcl10 the results of qPCR confirmed those obtained using CDS analysis with excellent quantitative agreement (compare Fig 4E to 4F). Of note is that some transcripts such as those for NPY were detected exclusively in the α7^E260A:G^ mice or in much greater relative abundance compared to the α7^G^ mice (not shown). While NPY protein expression was detected in the lung (Fig 2A), the possibility that this discrepancy in α7-genotype transcript abundance could be due to small contaminants, CDS sequence overlap with other more abundantly expressed transcripts, and/or detection of intervening LTRs adjacent to exons remains to be completely defined. Thus, in addition to CDS calculation, a second cut-off limited the analysis to those transcripts that exhibited more than 0.06 RPKM (Reads Per Kilobase of transcript per Million mapped reads) based upon an average total reads of the α7^G^ and α7^E260A:G^ response with or without LPS. For the present analysis this reflects a stringency of ~200 average read depth being required for any single experimental sample. Of 20564 possible transcripts in this analysis, approximately 6181 transcripts received no reads while 9084 transcripts (falling between a CDS average read-depth of 1 to 199) were removed. The remaining 6437 transcripts reached or exceeded these stringent cut-offs (see S1 Table). Of these the false-positive rate is estimated at approximately 0.3% as confirmed through validating the gene alignment of RNA-Seq reads to the UCSC Genome browser assembly NCBI37/mm9 (e.g., Fig 4E). The false negative rate is less certain although it is unlikely to exceed 1% based upon random sampling of “detected” transcripts and absence of reproducible signaling between independent replicates (not shown). Also, markers assigned to NE cells such as the expression of TH transcripts failed to reach significance despite the detection of this gene’s protein (Fig 2B). However, as was noted, cells expressing TH were rare and the very small cell number would mostly likely account for the lack of sensitivity required to examine the expression of this transcript further in the present study. The detection of many immunohistochemical neuronal marker transcripts such as CGRP or peripherin, which was observed only in axons, would not be likely since the cell bodies express the markers and hence their transcripts would not be present in our sampling. Finally, immunoglobulin, ribosomal and mitochondrial encoded genes were removed for clarity in this part of the analysis. As will be shown, however, this approach to analysis greatly reduced transcript noise and enhanced attention towards those signals of most relevance to the α7^E260A:G^ genotype-associated response in distal lung epithelium.

Finally, because many previous studies have applied the Affymetrix microarray platform to assess lung epithelium gene expression (e.g., [47–49]), we measured how the results obtained from RNA-Seq correspond to results obtained using that method. To summarize, the results from both platforms produced strong agreement as shown in Fig 4F although measurements using Affymetrix lack the dynamic range of RNA-Seq, producing a compressed distribution as would be expected. This comparison offers additional confirmation that the RNA-Seq results are reproducible when using a different analysis platform, and they support the ability to compare these findings with those from other studies that relied upon the Affymetrix array-based platform.

### The α7-coupled transcriptional networks suggest cell-specific responses

**Fig 5:**
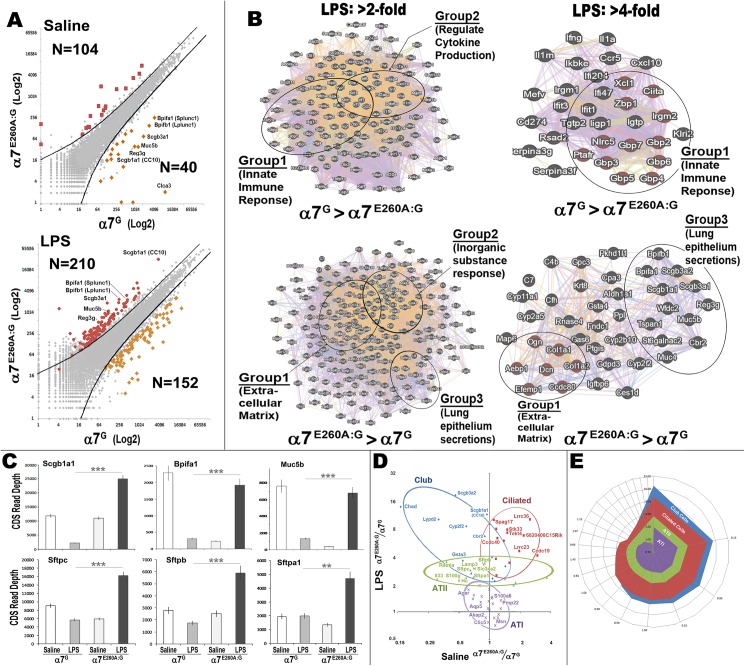
RNA-Seq results reveal α7-impact on cell-specific changes in transcription following LPS challenge. A) CD45^-^ interstitial cells were isolated and RNA-Seq performed. Transcript data were converted to CDS values and these were then compared as labeled between α7-genotypes following challenge with i.n. saline or i.n. LPS as indicated. The lines indicate a 2-fold threshold difference in expression between genotypes and the number (N) of gene transcripts exceeding the 200 average read depth minimal cut-off (see S1 Table). Genes achieving a 4-fold or greater are colored, and some of these genes that exhibit among the greatest change in relative expression between control (Saline) and i.n. LPS (LPS) treatments are identified by their gene name. B) GeneMANIA derived plots [23,24] based upon gene transcript read averages between α7-genotypes in response to i.n. LPS. Gene clusters to the left include transcripts exceeding a genotype-based average read depth of 2-fold or 4-fold expression for the gene clusters to the right. Diagrams were generated using the default settings (Max resultant genes and attributes were set to zero). Subsets of key functional gene groupings as defined by GeneMANIA are indicated. In the α7G control the i.n. LPS response is dominated by two highly significant functional groups inclusive of ‘innate immune response’ and ‘regulators of cytokine production’ gene sets. The ‘innate immune response’ groups are retained when the 4-fold stringency cut off analysis was applied. In contrast, the same analysis of the i.n. LPS response enhanced specifically in the α7^E260A:G^ lung epithelium reveals three different gene groups. These include genes of ‘extracellular matrix’, ‘inorganic substance response’ and ‘lung epithelium secretions’ of which the ‘extracellular matrix’ genes, and ‘lung epithelial secretions’ are retained after increasing stringency to greater than 4-fold. C) Quantitative average CDS read depth measures for each α7 genotype and treatment group are compared for some of the major genes defined both in the GeneMANIA analysis as epithelial secretions and from the plot in (A). The inverse relationship between gene expression in response to i.n. LPS that is related to α7^G^-genotypes is apparent and highly significant (** = p>0.01; *** = p>0.0001). D) Genes were subgrouped into defined cell-specific transcripts for Club (blue), ciliated (red), ATI (violet) and ATII (green) cells (Table 3 and S1 Table). The relative shift in α7^E260A:G^ expression after LPS is shown and some genes are identified that exhibit particularly robust shifts in expression by Club and ATII cells (relative increase by α7^E260A:G^ to the control) versus genes transcripts that were increased in ciliated cells for the same comparisons but relatively unchanged for ATI cells as shown by their grouping around 1.0 when compared to the expression differences in saline exposed (control tissues). E) Polar plots of the same cell-specific genes plotted in order of the difference in expression between saline and LPS exhibits the most dysregulation in Club cell gene expression followed by ciliated cells and ATII cells. ATI cells, which exhibit no α7-expression or number differences between genotypes, were again grouped around the expected 1.0 coordinates indicating no change in expression.

Comparisons between the α7^G^ and α7^E260A:G^ i.n. LPS transcriptional response as measured using RNA-Seq are summarized graphically in Fig 5A. Genes exhibiting a 2-fold or greater difference between α7^G^ and the α7^E260A:G^ i.n. saline (control) samples include 144 gene total transcripts of which 40 genes were consistently expressed more in the α7^G^ and 104 genes were expressed more in the α7^E260A:G^ (Table 1 (see S1 Table) and Fig 5A). These values were increased after i.n. LPS to 362 total genes exceeding a 2-fold difference between α7 genotypes. This included 152 genes expressed preferentially in the α7^G^ samples and 210 in the α7^E260A:G^ (Table 2 (see S1 Table) and Fig 5A). Some examples of the genes that were reduced in constitutive expression in the α7^E260A:G^ include Postn (periostin), whose gene product imparts cell adhesion and extracellular matrix remodeling [50], Retnla (resistin-like alpha), which impacts on IL-6 secretion and allergic inflammatory responses [51,52], Cyp2a5 (cytochrome P450 family 2 subfamily a5) that modulates the LPS response and has been suggested to degrade nicotine [53], and Ltf (lactotransferrin) which participates in antimicrobial activity [54,55]. Also, a recurring gene that is suppressed in the α7^E260A:G^ is Lcn2 (lipocalin2), which can also contribute to modulating the host defenses to multiple bacterial species [54,55]. One striking aspect of the difference in gene expression between these genotypes is revealed after i.n. LPS. As highlighted for a subset of these genes in Fig 5A, their expression was substantially greater in the α7^G^ versus α7^E260A:G^ lung, but completely reversed in terms of their response and expression levels following i.n. LPS. Some of these are identified by gene name in Fig 5A and they are recognizable as genes important to lung secretory and epithelial function. This issue is returned to in greater detail below.

**Table 1:** Saline α7^E260A:G^ versus α7^G^ Response Difference (Gene Ratio >2-fold).

Gene (E>G)	Fold Change	(continued) (E>G)	Fold Change		Gene (G>E)	Fold Change
Gdpd3	26.6	Fam108c	2.3		Clca3	2373.5
St6galnac2	8.9	Slpi	2.3		Muc5b	21.2
Saa3	7.6	Abcd2	2.3		Reg3g	16.1
Marco	6.1	Sh3pxd2b	2.3		Scgb3a1	15.6
Map6	5.0	Il1a	2.3		Bpifb1	11.8
F10	4.9	Mfhas1	2.3		Bpifa1	10.0
Selp	4.8	Tnf	2.3		Cyp2a5	5.3
Crispld2	4.5	Steap4	2.3		Tfdp2	3.5
Prg4	4.3	Wfdc17	2.3		Gp5	3.2
C4b	4.2	Cebpa	2.2		Gsr	2.9
1100001G20Rik	3.9	Fabp1	2.2		Retnla	2.7
Gpnmb	3.8	Anxa4	2.2		Klri2	2.6
Il1f9	3.7	Inppl1	2.1		Postn	2.6
Fabp5	3.7	Pvrl3	2.1		Iigp1	2.5
Rab20	3.4	Atp6v0d2	2.1		Ifi47	2.5
Alox15	3.4	Ms4a8a	2.1		Gsto1	2.5
Fgfr1	3.4	Ltbp1	2.1		Lama4	2.5
Msr1	3.4	Frrs1	2.1		Hspa1b	2.5
Slc39a2	3.3	Trim29	2.1		Tgtp2	2.5
Serpinb2	3.3	1600029D21Rik	2.1		Ltf	2.4
Slc40a1	3.2	Lrg1	2.1		Isg15	2.4
Wls	3.0	Csf3r	2.1		Pttg1	2.3
Cxcl10	3.0	Rufy4	2.1		Ifit1	2.3
Ccdc80	3.0	Hebp1	2.1		Gp9	2.3
Tbc1d2	2.9	Mrc1	2.1		Oasl2	2.2
Gdf15	2.8	Il1rn	2.1		Slc4a1	2.2
Kcnn3	2.8	Dhrs7	2.1		Scgb3a2	2.2
Acaa1b	2.7	Sgms2	2.1		Ciita	2.1
Ptgis	2.6	1810033B17Rik	2.1		Igtp	2.1
Padi4	2.6	Mmp9	2.0		Mpl	2.1
Clec4e	2.5	Ltc4s	2.0		Gp1ba	2.1
Serpine1	2.4	Abi3bp	2.0		Gm12250	2.0
Arg2	2.4	Mpp6	2.0		Cdr2	2.0
Serpinb9b	2.4	Cyp26b1	2.0		Mgp	2.0
Slc9a4	2.4	Il1b	2.0		Mfsd2b	2.0
Dab2	2.4	4930506M07Rik	2.0		Lipg	2.0
Clec4n	2.4	C2cd2l	2.0		Alox12	2.0
Lilra5	2.4	Dst	2.0		Pla2g16	2.0
Il1r2	2.4	Gpr137b	2.0		Hspa1a	2.0
Fabp4	2.4	C1qb	2.0		Bpgm	2.0
Bst1	2.3	Mmp8	2.0			
Slc7a2	2.3	Nrp2	2.0			
Siglece	2.3	Ctsd	2.0			
Fn1	2.3	Ly75	2.0			
Glrx	2.3	Clec4d	2.0			

**Table 2:** LPS α7^E260A:G^ versus α7^G^ Response Difference (Gene Ratio >2-fold).

Gene (E>G)	Fold Change	(cont) (E>G)	Fold Change		Gene (G>E)	Fold Change	(cont) (G>E)	Fold Change
Scgb3a2	18.7	Slco2b1	2.5		Gbp5	25.0	Pyhin1	2.9
Scgb1a1	11.5	Hgf	2.5		Cxcl10	18.8	Klra7	2.9
Bpifb1	10.8	Cgnl1	2.5		Ifng	13.7	Niacr1	2.9
Pkhd1l1	10.7	Glb1l2	2.5		Gbp4	10.5	Casc4	2.9
Gdpd3	10.3	Cdh1	2.5		Gbp6	10.4	Psmb9	2.9
Dcn	9.9	Krt79	2.5		Gbp2	9.3	Slc7a11	2.9
Reg3g	9.3	Mmp15	2.5		Il1a	9.0	Herc6	2.9
Aebp1	9.2	Matn2	2.5		Tgtp2	8.9	Pydc3	2.9
Efemp1	8.9	Il7r	2.5		Il1rn	7.7	Bub1b	2.9
Gpc3	8.9	C77080	2.5		Serpina3g	7.5	H2-T24	2.6
2410076I21Rik	8.7	Mettl7a1	2.5		Iigp1	7.3	Prf1	2.5
St6galnac2	8.7	Cyp1a1	2.5		Gbp3	6.9	Il2rb	2.5
Cyp2f2	8.6	Slc4a1	2.4		Ifi204	6.9	Fcho1	2.5
Cyp2a5	7.9	Cidec	2.4		Ikbke	6.7	Ccl5	2.5
Gsta4	7.8	Mgst1	2.4		Ifit1	6.7	AW112010	2.5
Igfbp6	6.9	Col18a1	2.4		Gm12250	6.3	Klrk1	2.5
Cbr2	6.7	Ppt1	2.4		Igtp	6.0	Irf7	2.5
Bpifa1	6.3	Gprc5a	2.4		Serpina3f	6.0	Bcl2l11	2.1
Scgb3a1	6.1	Sftpa1	2.4		Ciita	5.7	Nr4a2	2.1
Sec14l3	6.1	Ltbp4	2.4		Ifi47	5.3	Icam1	2.1
Cpa3	6.0	Pex14	2.4		Zbp1	5.1	Sord	2.1
C7	5.8	Fabp5	2.4		Irgm2	5.0	Bcl2a1b	2.1
Cyp11a1	5.8	Siglecg	2.3		Irgm1	4.9	Asprv1	2.1
Tspan1	5.7	Inadl	2.3		Gbp7	4.9	F5	2.1
Wfdc2	5.6	Alox15	2.3		Cd274	4.9	Nr4a3	2.1
Map6	5.4	Cks2	2.3		Ccr5	4.7	Parp12	2.1
Cfh	5.3	Top2a	2.3		Xcl1	4.5	Malt1	2.0
Muc5b	5.2	Fbn1	2.3		Mefv	4.2	Klre1	2.0
Rnase4	5.1	Fn1	2.3		Nlrc5	4.2	Gbp8	2.0
Ccdc80	5.0	Mpp6	2.3		Ifit3	4.2	Zap70	2.0
Cyp2b10	4.8	Tacc2	2.3		Ptafr	4.1	Ptprcap	2.0
Krt8	4.8	Rgs1	2.3		Klri2	4.1	Ube2f	2.0
Aldh1a1	4.7	C3ar1	2.3		Rsad2	4.1	Bhlhe40	2.0
Muc4	4.7	Pllp	2.3		Irf4	3.8	Atp2b4	2.0
Col1a2	4.5	St3gal5	2.3		Dtx3l	3.8	Dll1	2.0
Ptgis	4.5	Tnfrsf21	2.2		Isg15	3.8	Gadd45b	2.0
Col1a1	4.5	Trerf1	2.2		Rtp4	3.7	Adora2a	2.0
Fndc1	4.4	Entpd5	2.2		Klrb1b	3.6	Nfkbib	2.0
Ppl	4.4	Lsr	2.2		Gbp9	3.6	Il1b	2.0
Ces1d	4.4	Amz1	2.2		Upp1	3.6	Ms4a6b	2.0
Ogn	4.2	Kcnn3	2.2		Pttg1	3.6	Adamts14	2.0
C4b	4.2	Inmt	2.2		Stat1	3.6	Irf5	2.0
Gas6	4.0	Pvrl3	2.2		Irg1	3.5	Ifi27l2a	2.0
Col3a1	3.8	Hebp1	2.2		Klra9	3.5	Dusp2	2.0
Gsn	3.7	BC003331	2.2		Irf1	3.4	Trim12c	2.0
Retnla	3.5	Surf2	2.2		Tap1	3.3	Ptpn4	2.0
Gsto1	3.5	Ltc4s	2.2		Cfb	3.3	Parp9	2.0
Serping1	3.5	Comt	2.2		Gsr	3.2		
Nat6	3.4	Dock7	2.2		Ccrl2	3.2		
Enpp2	3.4	Spata6	2.2		Klrb1c	3.2		
St14	3.4	Pla2g4a	2.2		Nfkbie	3.1		
Sftpb	3.4	Igfbp7	2.2		Slfn8	3.0		
C1s	3.4	Ift140	2.2		Sod2	3.0		
Selenbp1	3.4	Pnpla7	2.2		Pim2	3.0		
Wls	3.3	Slc48a1	2.2		Emr4	3.0		
Cyp26b1	3.2	Cd200r4	2.2					
Synm	3.2	Lilra5	2.2					
Myh14	3.2	C1ra	2.2					
Higd2a	3.2	Rbx1	2.1					
Nupr1	3.1	Pnpla6	2.1					
Il6	3.1	Itfg3	2.1					
Fabp4	3.1	Ear1	2.1					
Krt19	3.1	Zrsr1	2.1					
Gcnt1	3.1	Pparg	2.1					
Cpne5	3.0	Galnt3	2.1					
Mapkbp1	3.0	Idh1	2.1					
Crip1	3.0	Egflam	2.1					
Lamp3	3.0	Stk11ip	2.1					
Csrp2	3.0	Cgrrf1	2.1					
Prom1	3.0	Ngp	2.1					
Slc34a2	3.0	Anxa1	2.1					
Zfp868	3.0	Tmed3	2.1					
Rab44	2.9	Pon3	2.1					
Pi16	2.9	Eif2b4	2.1					
Sftpc	2.8	Chi3l3	2.1					
Coro6	2.8	Ptch1	2.1					
Serpina3n	2.8	Cdk2	2.1					
Krt18	2.8	Mocos	2.1					
Snca	2.8	Card9	2.1					
Krt7	2.8	Zfp639	2.1					
Fgd2	2.8	Cbr1	2.1					
C3	2.8	Lama2	2.1					
Tmc4	2.8	Spns1	2.1					
Ube2l6	2.8	Cant1	2.1					
Asph	2.7	Il18	2.1					
Fech	2.7	Mesdc2	2.1					
Abcc3	2.7	Pla2g15	2.0					
Cp	2.7	Alox5	2.0					
Il8	2.7	Slc12a2	2.0					
Cebpa	2.7	Cxcr2	2.0					
Pgrmc1	2.7	Tppp3	2.0					
Ptprs	2.7	Il6ra	2.0					
Bpgm	2.6	Galns	2.0					
Igsf8	2.6	Tspan12	2.0					
Slc25a39	2.6	Sh2d1b1	2.0					
Ltbp3	2.6	Clec4a2	2.0					
L1cam	2.6	Oxct1	2.0					
Gsta3	2.6	Carkd	2.0					
Ednrb	2.6	Per2	2.0					
Col6a2	2.6	B3gnt7	2.0					
Pcolce	2.6	Cryzl1	2.0					
Ltf	2.6	Atp9a	2.0					
Gpnmb	2.6	Gpr126	2.0					
Ptprf	2.6							
Mdm1	2.6							
Dhx40	2.6							

To further examine how functional responses between the α7^G^ versus α7^E260A:G^ i.n. LPS could be altered, the overall expression profile of transcripts from Table 2 was visualized using the GeneMANIA analysis method (Fig 5B; [23,24]). This revealed α7-genotype unique groupings. For example, two major gene groups are disproportionally increased in the α7^G^ relative to the α7^E260A:G^. These include one gene group with strong association to the ‘innate immune response’ (21 genes; FDR = 1.09e-25) and a second group is associated with positive ‘regulation of cytokine production’ (11 genes; 3.76e-11). Some specific examples in these groups include the selective disproportionate increase by α7^G^ in multiple pro-inflammatory cytokines and chemokines (e.g., IL-1α, IL-1β, TNFα, IFNγ, Cxcl10). These also share an association with the Nfκb/Ifκb transcript family activation. The similar inflammatory response to i.n. LPS is not observed in the α7^E260A:G^ mouse. In fact, in contrast to the α7^G^ mouse, the α7^E260A:G^ responsive gene groups vary considerably from those identified in the α7^G^ analysis. As shown in Fig 5B the α7^E260A:G^ > α7^G^ response produced three major gene clusters that share function in defining the ‘extracellular matrix’ (22 genes, 2.5e-9) and a group classified as ‘response to inorganic substances’ (17 genes, 2.42e-6). The third group cluster is composed of transcripts associated with surfactant and mucin production. These genes are particularly dysregulated in expression after LPS when the α7-signaling is uncoupled to these modulatory processes as in the α7^E260A:G^ mouse. Thus, this analysis confirms earlier results [14] that the response to i.n. LPS as measured by pro-inflammatory cytokine and chemokine induction is reduced in the α7^E260A:G^ mouse. However, this finding extends this to include the epithelium response which further emphasizes that a strong component of the lung response to i.n. LPS includes a CD45^-^ cell fraction modulated by α7. When stringency of the input data to GeneMANIA is increased to allow only those genes differing in expression by greater than a highly significant 4-fold (Fig 5B), the α7^G^ Group1 ‘innate immune response’ genes are retained. A similar analysis of the α7^E260A:G^ response shows that genes encoding functional members of the Group 1 ‘extracellular matrix’ and Group 3 ‘lung epithelial secretions’ are retained when stringency is increased. These results reveal distinct shifts by the lung epithelium transcriptional response to i.n. LPS that is in these cells associated with α7 signaling.

As noted above and in Fig 5A, some of the genes are distinguished by their highly disproportionate quantitative transcript response to i.n. LPS. Among the genes expressed specifically by Club cells including members of the secretoglobins (e.g., Scba1a1 (CC10) and Scgb3a1), Splunc/Plunc family members (e.g., Bpifa1 and Bpifa2; [56]) and the mucin, Muc5b. The surfactant protein genes (Sftpc, Sftpb, and Sftpa1) are specific to ATII cells [57]. These are mostly in the “lung epithelium secretions” functional Grouping (Fig 5B). The quantitation for a representative subset of these genes and their responsiveness to i.n. LPS is shown in Fig 5C. As noted, for these genes there is a dramatic and inverse relationship between their control expression levels and their response following i.n. LPS which is collectively and substantially increased in the α7^E260A:G^. This transcriptional comparison was extended to determine if the altered α7-transcriptional responses are to lung epithelial cell subtypes. In other reports cell-specific gene expression profiles were assembled from RNA-Seq analysis of genetically-identified distal lung epithelium single cell isolates [57]. These include signature transcript sets for four distinct epithelium populations from both bronchiolar and alveolar sites including major transcripts that define Club, ATII, ciliated, and ATI cells. These cell-specific gene sets were compared to our results as shown in Table 3, or plotted in a principle component analysis format as shown in Fig 5D. In each case transcript differences between the different α7 genotypes and their respective response to i.n. LPS fall into distinct clusters defined by their origin from Club, ciliated, ATI and ATII cells, respectively. Both Club and ATII transcripts, which express α7, tend to be shifted towards enhanced expression to an i.n. LPS challenge in the α7^E260A:G^ cells. The disruption of Club cell gene expression is particularly robust. This includes the secretoglobins and the Cyp2f2, a cytochrome P450 involved in the Club cell response to naphthalene [58]. Other genes such as Chad (chondroadherin) and Lypd2 (Ly6/Plaur domain containing 2) produce products that are associated with the extracellular matrix. Also altered are multiple genes associated with ATII cells including the surfactant family genes Sftpa1, Sftpb and Sftpc. Other transcripts of interest include IL-33 that in mice is involved in host defense and wound repair [59], Hc (hemolytic complement) that contributes to the inflammatory response in allergic lung disease and Retnla (resistin-like alpha) that is also a contributor to attenuating allergic inflammatory responses [52]. The ATII cells exhibit expression variance that is most notable in the horizontal axis which is consistent with the strong tendency of the gene expression in these cells to be scaled in the direction of α7^G^ expression exceeding α7^E260A:G^ under constitutive conditions, but exhibiting a strong reversal of this relative α7-gentoyte expression difference in response to i.n. LPS challenge. In contrast, the ciliated cell transcripts form a distinct and more tightly localized cluster indicative of an overall quantitatively similar response to i.n. LPS between the different α7 genotypes. This would be consistent with their substantially reduced number in the α7^E260A:G^ and is further suggested by the expected shift in cilia-related genes such as the coiled-coil domain containing genes Ccdc40 and Ccdc19 and leucine rich repeat containing genes Lrrc36 and Lrrc23.

**Table 3:** Lung epithelium cell-specific transcript expression differences. (α7^E260A:G^ / α7^G^).

**Club**	**Saline**	**LPS**		**ATII**	**Saline**	**LPS**
***Scgb3a2***	0.46	18.67		***Retnla***	0.38	3.52
Chad	0.13	13.86		***Sftpb***	0.90	3.39
***Scgb1a1***	0.93	11.51		Lamp3	0.70	3.00
Lypd2	0.32	10.13		*Slc34a2*	0.72	2.99
Krt15	1.00	9.00		***Sftpc***	0.65	2.85
***Cyp2f2***	0.59	8.55		***Sftpa1***	0.69	2.38
*Cbr2*	0.90	6.65		Lgi3	0.59	2.36
Aldh1a7	1.06	3.92		*Fabp5*	3.66	2.35
1810010H24Rik	0.55	3.60		Etv5	1.41	2.28
Nupr1	1.43	3.14		*Il33*	0.31	2.26
*Gsta3*	0.66	2.61		S100g	0.50	2.23
Pir	1.07	2.31		**Chi3l1**	1.80	2.08
Dcxr	1.12	2.16		Lgals3	1.73	1.95
				Ager	0.80	1.93
				Emp2	0.99	1.76
				Akap5	0.62	1.74
				Hopx	0.70	1.52
				Dpysl2	0.84	1.47
				Lmo7	1.06	1.44
						
**Ciliated**	**Saline**	**LPS**		**ATI**	**Saline**	**LPS**
Lrrc36	2.50	10.13		S100a6	1.05	1.41
Spag17	1.10	10.00		Rtkn2	0.69	1.38
Wdr16	1.17	8.00		Pmp22	1.29	1.34
Stk33	1.50	7.83		Sdpr	0.98	1.31
Tekt4	1.50	7.25		**Aqp5**	0.82	1.28
6820408C15Rik	2.17	6.75		Cav1	1.08	1.23
Ccdc40	1.19	6.33		Ptrf	0.96	1.09
1700009P17Rik	1.38	5.93		Qk	1.29	1.08
Lrrc23	1.97	4.59		Agrn	1.00	1.05
Ccdc39	2.96	4.10		Ahnak	1.23	0.94
Foxj1	1.16	3.75		Akap2	0.91	0.87
Ccdc19	1.57	3.42		Msn	1.21	0.87
Kif9	1.17	2.48		Vegfa	1.11	0.86
				Clic5	0.97	0.77
				Timp3	1.14	0.71

ATI cell transcripts [57] exhibited little difference in their expression either related to α7-genotype or i.n. LPS. These include Aqp5 (aquiporin5) and Ager (advanced glycosylation end-product). This is also consistent with the similarity in ATI cells between α7-genotypes and their lack of tGFP expression. When these data are replotted as a polar projection (Fig 5E) to visualize the deviation for each cell-specific gene group from 1.0, the relative impact of α7 uncoupling to spread in variance of their cell signaling response is most apparent in the Club cell transcript group followed by the ciliated cells. The ATII cells exhibit dysregulated expression that exceeds the expectation of variance from 1.0 that is seen for ATI cells. Thus, this analysis supports that the impact of α7 on the LPS response in lung epithelium is a summation of the more direct impact of α7 modulation of gene expression in cells where it is expressed (Club or ATII) and scaling that reflects cell number differences between α7 genotypes.

### Disrupted α7-signaling corresponds with mucin accumulation and airway blockage

**Fig 6:**
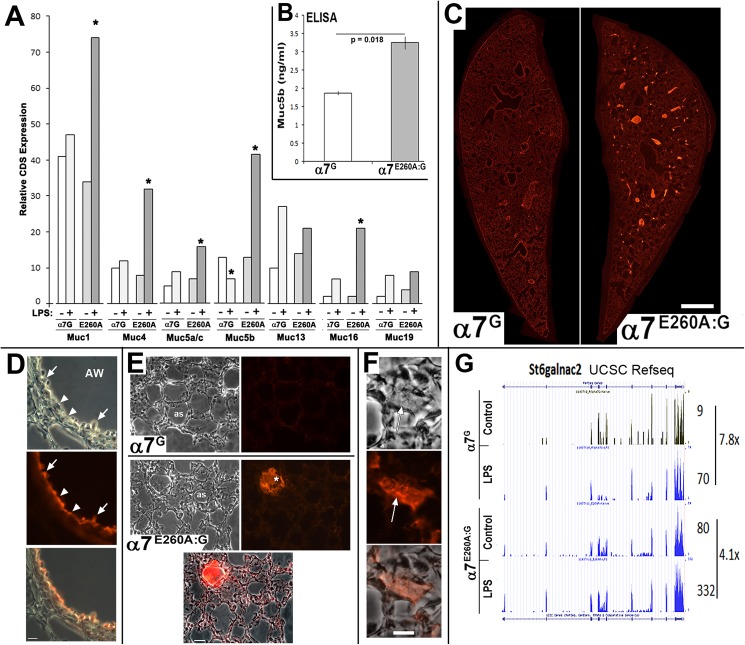
Dysregulation and accumulation of mucins in airways of the α7^E260A:G^ lung. A) RNA-Seq results showing CDS quantitation of read-depth for the identified mucins in sample of α7^G^ or α7^E260A:G^ (E260A) genotypes with (+) and without (-) i.n. LPS challenge. Asterisk (*) designates a significant (P<0.05) difference from the paired genotype control. B) ELISA measuring Muc5b in mouse BALF from 3 independent samples (in replicate) of each genotype. As predicted by the RNA-Seq results, Muc5b was elevated significantly in the α7^E260A:G^ compared to control samples. C) Lungs of each α7 genotype were fixed and prepared for immunohistochemical analysis as described in the Methods. The sections shown are longitudinal slices stained for immunoreactivity to Muc5b and visualized with a red fluorochrome. Immunostaining of bronchial pathways is apparent in the α7^G^ mouse. However, in the α7^E260A:G^ there are multiple deposits of Muc5b that coincide with many of these airway passages. D) Increased magnification of a bronchial lining of the α7^G^ lung reveals immunostaining towards Muc5b lining the bronchia (arrow heads) but also associated with Club cells (arrow), which in the mouse are a major source of this mucin. AW = airway. E) Comparisons of the expression of Muc5b in the α7^G^ (upper panels) and α7^E260A:G^ (lower panels). Phase microscopy images reveal alveolar spaces (as) and the same section stained for Muc5b is shown to the right (merged image is also shown for the α7^E260A:G^ section). The α7^G^ staining is low or not detected as would be expected in these sites that lack Club cells and Muc5b production. In the α7^E260A:G^ lung accumulations of Muc5b (asterisk) in similar spaces is seen and immunostaining in the matrix of the alveolar spaces appears to be enhanced over controls. F) Increased magnification of some sites of Muc5b accumulation in the α7^E260A:G^ mouse shows the fibrous appearance often associated with these bodies in the phase microscopy image (arrow). G) An example of changes in the expression of transcripts that encode proteins involved in post-translation modifications of mucins and other secreted proteins. Shown is the RNA-Seq CDS count (numbers to the right) for the gene St6galnac2 whose protein is alpha-N-acetylgalactosaminide alpha-2,6-sialyltransferase 2, an important O-linked glycosylation enzyme. In control α7^G^ lung this gene is expressed at relatively low levels that are increased almost 8-fold after LPS stimulation. In the α7^E260A:G^ lung the basal expression is already almost 9-fold over the α7^G^ lung and its expression increased an additional 4-fold in in response to LPS. This dramatic increase in overall St6galnac expression is similar to that observed for Muc5b and other proteins targeted by the gene product activity. Bars (microns) = C, 700; D, 30; E, 45; F, 15.

The expression of Muc5b, associated surfactants and secretory proteins as well as the increased production of extracellular matrix proteins between α7^G^ and α7^E260A:G^ lung epithelium as suggested by RNA-Seq was investigated further. The first analysis focused on the mucins. As shown in Fig 6A, RNA-Seq results revealed that in addition to Muc5B, the expression of other Muc transcripts in the α7^E260A:G^ are significantly altered (increased) in response to LPS (notably Muc1, Muc4, Muc5a/c, Muc16). As noted, Muc5a/c expression was included in the mucin transcripts that were altered by α7 genotype similar to the other mucins despite the relatively low level of transcript expression [60].

In the mouse Muc5b is largely expressed by Club cells lining the bronchi and examination of its protein expression also reveals abnormal expression. This is seen in the BALF where ELISA analysis for Muc5b protein shows the α7^E260A:G^ contains an increased constitutive Muc5b protein amount relative to the α7^G^ mouse (Fig 6B). Muc5 protein expression in unstimulated α7^G^ and α7^E260A:G^ mice is consistent with this result as revealed by immunohistochemistry of the lung (Fig 6C). As seen there, the localization and relative density of Muc5 protein signal is drastically different between mice of the different α7-genotypes. Whereas overall the staining for Muc5 in the α7^G^ lung is relatively weak and mostly restricted to bronchial passage lining (consistent with Club cell location), in the α7^E260A:G^ mouse it was readily detected in 6 of 9 of the animals examined. As shown, the Muc5 reactivity presented as a substantial build-up that often filled a portion of the distal bronchia and bronchioles (Fig 6C). At greater magnification this expression pattern in the α7^E260A:G^ lung was confirmed to correspond with Club cells and the immunoreactivity was dispersed along the bronchial linings of unobstructed air way passages. Similar staining in the α7^G^ mouse was much weaker and in both α7 genotypes it did not extend into the distal alveolar structures (see Fig 6E). There was, however, increased generalized staining for this protein particularly in the interstitial regions where in the α7^E260A:G^ lung additional accumulation was evident (Fig 6E). The increased magnifications view of these smaller accumulations reveals their fibrous appearance (Fig 6F). The differences shown are particularly striking because they occur in the absence of any treatment, and despite the generally reduced expression of the Muc5b gene in the α7^E260A:G^ mouse in the absence of LPS stimulation, perhaps reflecting the reduced number of ciliated cells and consequently compromised removal. Although these accumulations were not necessarily worsened immediately following i.n. LPS (not shown), we anticipate further severe build-up of mucins in the α7^E260A:G^ mouse could be present as the time after LPS is increased before analysis.

The association of Muc5b expression to Club cells in the mouse again indicates dysfunction in this cell type in the α7^E260A:G^ mouse. In this context there is also parallel dysregulation of the expression of several genes important to post-translational modification of Muc5 proteins. This is shown for one of them, the mucin glycosylation gene St6galnac2 (Fig 6G) and gene product that functions as a sialyltransferase to add terminal sialic acids to glycoconjugates on proteins including mucins [61]. Like Muc5b, and other mucins, this gene transcript in α7^E260A:G^ samples is highly elevated both constitutively and following i.n. LPS where its expression is almost 5-fold over the α7^G^ control. This result suggests that in the α7^E260A:G^ mouse, high end glycosylation capacity appears elevated and increases similarly to the mucins whose expression is altered after inflammogen challenge.

### The α7^E260A:G^ lung exhibits increased expression of extracellular matrix gene expression and extracellular matrix deposits

**Fig 7:**
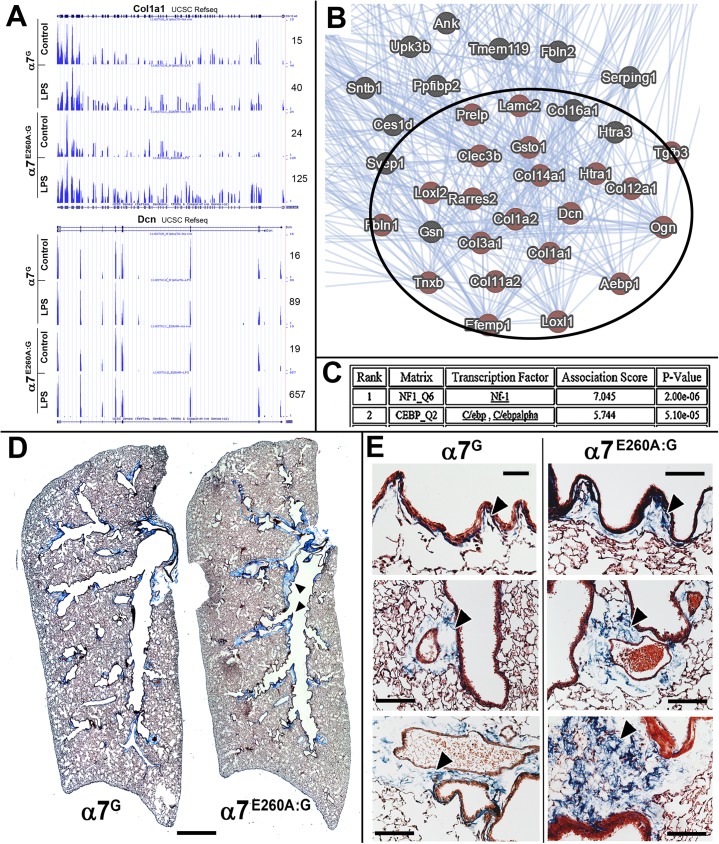
Disproportionate expression of fibrotic genes in the α7^E260A:G^ and accumulation of connective-like tissue. A) The results of RNA-Seq reveal enhanced expression in the α7^E260A:G^ lung and disproportionate increases in their response to LPS relative to controls. Examples of this are shown for Col1a1 and the decorin gene (Dcn) whose gene product binds to secreted collagen and contributes to crosslinking of the extracellular matrix. These genes are particularly overexpressed in the α7^E260A:G^ after challenge by LPS as reflected by the nearly 7-fold increase in expression relative to the α7^G^ lung epithelium. B) When genes associated with the extracellular matrix exhibiting dysregulated expression (also see Fig 5B) were analyzed using Genemania, a strong interactive association is observed. C) Analysis of possible transcription factors, in-common among the dysregulated genes, using the PASTAA analysis program suggests a strong affiliation with NF1 (neurofibromin1) and the CCAAT-enhancer binding protein (C/ebp, C/ebp-alpha) family. D) Masson’s trichrome staining of whole lung mounts from an α7^G^ or α7^E260A:G^ age and gender matched mice. The α7^E260A:G^ mouse exhibits notable accumulation of connective tissue along bronchial airways (arrowheads) that is not present or greatly reduced in the α7^G^ lung. E) Increased magnification of similar sections as in D show the appearance of enhanced Masson’s trichrome staining comparing fibrotic-like depositions (arrow heads) at multiple sites in the α7^G^ to the α7^E260A:G^ lung. Deposits are normal but greatly reduced in the α7^G^. Bars (microns) = D, 700; E, 100

Dysregulated transcript expression by the α7^E260A:G^ includes a substantial increase in expression of a subset of collagen and other extracellular matrix genes (Table 1) after i.n. LPS (Table 2). As an example, this is reflected by the collagen gene, Col1a1, and other genes whose products regulate collagen fibril assembly such as Dcn (decorin; Fig 7A). These genes also formed one of the high probability clusters shown in Fig 5B to be dysregulated between the α7-genotypes when expanded and identified in a GeneMANIA generated diagram shown in part in Fig 7B. The possibility that this gene grouping is subject to similar transcriptional regulatory interactions are also noted. As shown for the results of PASTAA analysis of this gene grouping (Fig 7C), there is a reasonable propensity towards common regulation by NF1 and C/ebp, C/ebp alpha. This suggests that a common transcriptional regulatory pathway modulated by α7 could be present in the lung epithelial cells.

The increase in pro-extracellular matrix gene expression, suggestive of altered extracellular matrix proteins, was examined in longitudinal whole lung sections of 6 month old α7^G^ or α7^E260A:G^ mice (4–5 each) stained with the Masson’s trichrome method. This method is particularly sensitive for detection of collagen deposits (blue) and keratin in red. Examination of the lung sections revealed a substantial increase in extracellular matrix in the α7^E260A:G^ mouse that is particularly evident in association with proximal bronchioles. Increased magnification of matched lung regions also reveals local accumulations of matrix proteins in the α7^E260A:G^ mouse that are nearly absent or greatly reduced in the α7^G^ lung. Examples of these sites of excessive accumulation are shown in Fig 7E. Most notable are the increases in matrix immediately below the lining of the proximal bronchial space (Fig 7D) and in regions adjacent to more distal bronchial regions usually in association with blood vessels (Fig 7E). In all cases the fibrils deposited appear as aggregates that do not form regular linear arrays nor is a fibroblast change associated with the α7-genotype detected. Thus, under normal conditions and in the absence of known related inflammatory challenge the α7^E260A:G^ lung exhibits signs of increased susceptibility to fibrotic deposits that is predicted by the RNA-Seq profile.

## Discussion

There is strong clinical, genetic and pharmacological evidence showing that nicotine, acting through the ionotropic nAChR alpha7 (α**7**), alters inflammatory responses which in peripheral tissues is most typically an anti-inflammatory effect [1–5]. The function of α7 is best characterized as pleiotropic and cell type specific. For example, in neuronal cells α7 modulates presynaptic neurotransmitter release through its sodium/potassium current, but in non-neuronal cells the exceptional permeability of α7 to calcium ions [4] engages calcium-mediated cellular processes including Creb, PI3K, Jak2 and NF-κB [3,4,62–64]. To distinguish between α7 signaling mechanisms, we used a genetic approach and homologous recombination to generate *Chrna7* reporter mice [12–14]. This method of introducing a bi-cistronic reporter cassette results in minimal perturbation to the genomic context, copy number and gene regulatory context of *Chrna7*. This is also a significant advancement over the α7^KO^ or other transgenic models where the impact by the modifications can be masked by compensatory mechanisms or themselves produce unanticipated consequences. Further, the direct measurement of α7-transcript expression is accomplished through following the tGFP reporter, which corresponds well with receptor expression [12]. Thus applying an immunohistochemical analysis reveals that α7 is prominently expressed by AMs, and in the epithelium by epithelial Club and ATII cells. This would suggest a direct role of α7 in these cells upon interaction with nicotine or other ligands of this receptor (e.g., endogenous acetylcholine and/or choline). Further, as reported previously, the overall impact by the α7^E260A:G^ mutation on the mouse lung response to i.n. LPS is to diminish the inflammatory response in part through interfering with the lung’s ability to recruit cells from the blood into the tissue. In part this reflects a significant component of the α7-modulated response is signaled through the lung epithelium, which is an active participant in coordinating the innate immune response [14,65]. This is particularly evident for standard cytokines and chemokines such as epithelial derived IL-1β which together with other early response genes exhibit a dramatic absence of expression in the α7^E260A:G^ mouse subsequent to challenge by i.n. LPS. Collectively these results support the conclusion that α7 expressed by lung epithelium cells also regulates the innate inflammatory response through effects on signaling inflammatory cell trafficking into this tissue.

One mechanism through which α7 modulates the inflammatory response is in response to vagal nerve release of acetylcholine [1–3]. This is the mechanistic underpinnings to the ‘cholinergic anti-inflammatory pathway’ and is sufficiently robust to warrant ongoing clinical trials of drugs that target α7 function for therapeutic efficacy in a variety of inflammatory disorders [5]. However, vagal innervation is not necessarily required for this anti-inflammatory effect since the skin, which does not receive this innervation, also exhibits α7 anti-inflammatory effects that are in part signaled through keratinocytes [3,6–9]. Further, often overlooked is that physiological levels of choline can also serve as an agonist to α7 receptor function [4,66]. This points to an α7 interface to cell responses that in the context of the local environment proceeds both in concert with, but also independently of, parasympathetic innervation. The lung is an important target of vagal innervation, but how this controls the inflammatory response is not fully defined. Our results show α7-expression in local neuroendocrine cell clusters and possibly peripherin-labeled processes. This is not entirely unexpected since α7 is expressed in the norepinephrine expressing endocrine cells of the adult adrenal gland [67] and this receptor has multiple direct impacts on autonomic responses including the baro-response [68,69]. Thus, the expression of α7 by subsets of neuroepithelial or neuroendocrine cells would extend the modulatory role of this receptor to include local responses ranging from stretch to acute hypoxia. Finally, the apparent interaction between peripherin-positive nociceptive afferents and AMs associated with the bronchial lining, which again is particularly strong in the α7^E260A:G^ lung, is of interest and this supports a model where α7 plays a very specific role in modulating macrophage responses. Defining how lung innervation and interaction with a distinct subclass of AMs, and other NE-associated interactions, impacts the cell responses to foreign substances will be an important aspect of understanding how this receptor contributes to fine-tuning of inflammatory stasis and how nicotine use will alter long-term outcomes and susceptibilities to pathology including fibrosis (returned to below).

In this study we find that α7 signaling has a heretofore unrecognized and significant impact on epithelial cells both constitutively and in response to i.n. LPS (see Table 3 and Fig 5). Included in the gene groups affected are those that are closely associated with lung secretory activity In this category, the prominent dysregulation of gene expression by the α7^E260A:G^ lung includes Scgb1a1 (CC10), Muc5b and surfactant genes Sftpa1, Sftpb, and Sftpc as well as Bpifa1 (Splunc) and Bpifb1 (Plunc). These genes are expressed either prominently or exclusively by Club cells (e.g., Scgb1a1 (CC10), Muc5b and Bpifa1) or ATII cells (e.g., Sftpa1, Sftpb and Sftpc), both of which also express α7. This is particularly interesting because establishing a link between alterations in α7 signaling and corresponding changes in the expression of these genes in response to inflammatory stimuli has important implications towards understanding how long-term health will be impacted upon by chronic exposure to ligands of α7 including nicotine.

In humans the blockage of airways is associated with changes in mucin expression and changes in expression or retention of additional lung associated proteins including mucins (notably Muc5b; [70]), surfactant protein C [71] and sialyltransferases that contribute to both cell-cell interactions and bacterial adhesion [72,73]. Thus, in addition to airway-blockage, the normal local environment promotes the susceptibility to ongoing respiratory infections. This latter point is demonstrated in those with mutations in the MUC5B promoter (variant rs35705950; [70,74]}) or surfactant proteins that increase protein retention through increased production to promote susceptibility to interstitial pneumonia and eventually idiopathic pulmonary fibrosis [74]. Further, in human cases of increased expression of Muc5b, the gene product itself impairs cilium function to further reduce clearing; an effect that eventually impacts upon distal lung cell remodeling and changes in ciliated cell numbers. The α7^E260A:G^ lung epithelial expression of secreted proteins including Muc5b and surfactant proteins is of particular interest since mucin accumulation and airway plug formation proceeds despite overall reduced expression of these genes unless exposed to i.n. LPS. This argues that the role of α7 modulation of these processes is not entirely straightforward and it extends beyond signaling control of acute responses to include additional functions. In the mouse there is strong evidence of ciliated cell production from a common Club cell precursor [46], which is notable in the α7^E260A:G^ lung where Club cells are increased and ciliated cell numbers diminished. In this context α7 expressing (tGFP+) cells are also located adjacent to neuroendocrine cells identified by NPY-labeling. The location of these cells is consistent with the highly restricted Club-like cell subpopulations whose coincident location contributes to their retention of self-renewal capacity as well as restoration of damaged epithelium including ciliated cells [46,75]. Thus it is not unreasonable to suggest that in addition to uncoupling α7-modulatory control of gene expression in Club and ATII cells, where it is expressed, combined with deficiencies in ciliated cell numbers combine to create poor clearance of these secretory products and participates in their accumulation in airway spaces.

A second striking shift in α7^E260A:G^ transcriptional stasis was associated with the altered extracellular matrix gene expression and constitutive increases in fibrotic deposits (as detected using Masson’s staining) in linings of the more proximal bronchia. The reason for increased extracellular matrix deposits in the α7^E260A:G^ lung is not yet defined, however it would be expected to worsen with chronic inflammatory challenge such as produced by i.n. LPS and the corresponding increase in the expression of these genes. Often fibroblasts are responsible for collagen and related protein production [76]. However, extensive analysis of these cells in immunohistochemical sections did not suggest any obvious abnormalities such as a difference in their distribution or numbers. While others [77] have reported α7 expression in lung fibroblasts, we did not detect the tGFP α7 expression reporter in these cells (not shown). Our genetic approach to define α7 transcript expression provides heretofore unavailable precision and sensitivity towards identifying those cells that do, and do not, express α7. This is a substantial advancement over methods that rely on antibodies of unclear sensitivity and reliability (e.g., see discussion in [2,29]). While there is no direct measure of α7 expression in fibroblasts, our studies do not necessarily disagree with conclusion of studies demonstrating the effects of nicotine on lung fibroblasts [77] and there are additional considerations. For example the possibility of functional compensation in the α7KO by other nicotinic receptors is always an ongoing issue since this mouse exhibits the complete lack of receptor expression, an issue that at least in part is addressed in the α7^E260A:G^ mouse. Also, in another report [78] protein analysis of fibroblasts shows protein for nAChRs α4, α9, α10 and β2 but not α7, while the presence of α7 mRNA is discussed. These authors suggest that α4 is a major mediator of nicotine’s effects on lung fibroblasts. Another report [79] finds α7 expression in cultured human fibroblasts, although our experience is that culturing cells in vitro can alter normal expression patterns of these receptors. Finally, the possibility that fibroblasts are influenced by α7 indirectly, perhaps similar to altered ciliated cell number or signaling interactions, is certainly possible. For example a reduced pro-inflammatory response to i.n. LPS is likely to accompanied by a coincident increase in alternatively activated macrophages [80]. These macrophages signal repair mechanisms that when over-expressed increase transcriptional programs leading to fibrotic pathologies such as observed in the α7^E260A:G^ lung. This would include rapid progression when recurrent challenge enhances secretion of pro-fibrotic cytokines such as transforming growth factor beta 1 (e.g., [70,74,81,82]). The AM and other macrophage transcriptional profiles were not examined, but previous studies [13,14] suggest a possible preference by the α7^E260A:G^ mouse toward this alternative-activated macrophage state. This is also similar to the results of human studies examining idiopathic pulmonary fibrosis (IPF; [74,82]) where genetic-based overproduction of Muc5b and surfactant proteins (e.g., SftpA1and SftpC) contributes a strong muco-ciliary component in the cycle of dysfunctional response/repair leading to pathology that bears a remarkable similarity to the α7^E260A:G^ mouse.

We find that AM but not interstitial macrophages (IM) strongly express the α7 reporter (tauGFP). This expression difference suggests the heterogeneity of the macrophages, and similar lineage derived cells, present in the lung. The AM macrophage is the most prevalent immune cell in the alveolar spaces of the lungs and these cells have been the topic of much discussion regarding their origins and longevity. We (14) and others [83,84] have demonstrated that they are replenished upon bone marrow transplantation, as are the IM. Thus they can both originate from bone marrow derived precursors. However, the AMs occupy a distinct niche of the alveolar spaces and they are less phagocytic than IM thus harboring a very different inflammatory potential. Also, within our IM population other bone marrow derived myeloid cells are present [83]. For example, dendritic cells (DC) add another level of complexity to these studies. DC are often grouped into classifications of resident DC, mono-derived DC, and plasmacytoid DC which can also be distinguished by cell surface markers. Future studies will provide answers as to whether, within our defined IM classification, DC populations are also present in both the α7^G^ and α7^E260A^ mice and determine the contribution of these cells to the inflammatory and immune response generated in these mouse strains.

The extension of these results to cigarette-smoking (CS) is complicated by the cadre of additional bioactive compounds in CS as well as the influence of other impacts such as hypoxia. These would almost certainly be expected to overwhelm the effects produced by α7 alone. Also, nicotine can target multiple nAChRs in addition to α7 including the extended effects through α3 and α4 receptors. Thus, in this study the impact of α7 that is being measured is limited to the LPS response and probably does not fully extend to CS (although it still could be a component of the response). Thus, the damage being produced is limited to only this current model of LPS-α7 interaction, and this may in fact be very different when additional and different cells become involved as induced by differing challenges.

## Conclusions

The α7^E260A:G^ model reveals that α7 plays an important modulatory role in the lung epithelial response to LPS. When this receptor is uncoupled from endogenous signaling mechanisms there is a corresponding change in gene expression stasis and the response by specific cell types to acute i.n. LPS. This suggests that exposure to nicotine (or other agonists of α7) through smoking, or by direct inhalation of nicotine (vaping), will also influence the susceptibility to environmental challenges that induce inflammation (e.g., particulates, allergens, etc) and affect the progression of the response. Importantly, we are optimistic that this can provide an accessible experimental model that will permit both the examination of mechanisms underpinning disease processes and how nicotine imparts its influence on them.

## Supporting information

S1 Table Lung epithelium RNA-Seq results summary. The gene transcript average read depth for those genes exceeding 200 mean counts between lung distal epithelium samples from the α7^G^ or α7^E260A:G^ exposed to intranasal saline or intranasal lipopolysaccharide. Also shown are ratios between sample results as indicated. For these data all immunoglobulin and ribosomal genes were also omitted.
(XLSX)
